# Analysis of the Economic Burden of Chronic Kidney Disease With Comorbidities Among Patients in Xuzhou, China

**DOI:** 10.3389/ijph.2024.1607000

**Published:** 2024-07-04

**Authors:** Wan Jie, Minghong Yao, Mingqi Wang, Yuning Wang, Yulong Jia, Yanmei Liu, Kang Zou, Xin Sun

**Affiliations:** ^1^ Institute of Integrated Traditional Chinese and Western Medicine and Chinese Evidence-Based Medicine Center and Cochrane China Center and MAGIC China Center, West China Hospital, Sichuan University, Chengdu, China; ^2^ NMPA Key Laboratory for Real World Data Research and Evaluation in Hainan, West China Hospital, Sichuan University, Chengdu, China; ^3^ Sichuan Center of Technology Innovation for Real World Data, West China Hospital, Sichuan University, Chengdu, China

**Keywords:** chronic kidney disease, comorbidity, economic burden, medication patterns, composition of expenses

## Abstract

**Objectives:**

To analyze the costs and medication patterns of patients with chronic kidney disease (CKD) and comorbidities in Xuzhou, China, using a large electronic medical records database.

**Methods:**

Data were obtained from an electronic medical records database. The annual per-person and per-visit cost of hospitalization, as well as the proportions of those costs, are presented.

**Results:**

The majority of the participants were middle-aged men, and had medical insurance. Glomerulonephritis was the primary cause of CKD in patients with an identified etiology. The average per-visit cost of hospitalization for the CKD-renal anemia and CKD-mineral and bone disorder groups was 8,674.5 (5,154.3–13,949.6) and 8,182.6 (4,798.2–12,844.7) Yuan, respectively, which was greater than that of the other groups. The major expenses incurred were for diagnostics, drug usage, surgical procedures, laboratory tests and material costs.

**Conclusion:**

The substantial burden imposed by CKD with comorbidities indicates the importance of implementing public health strategies aimed at detecting and preventing these conditions in the general population. With the aging population, our nation may experience a greater CKD-related economic burden.

## Introduction

Chronic kidney disease (CKD) is a significant cause of mortality and disability, making it a prominent public health issue worldwide [1, 2]. CKD is defined as kidney damage or an estimated glomerular filtration rate (eGFR) < 60 mL/min/1.73 m^2^ for more than 3 months. Kidney damage is defined as either functional abnormality of the kidneys or structural abnormalities on imaging studies. The estimated prevalence of CKD is 13.4% globally and the number of patients requiring renal replacement therapy for end-stage renal disease (ESRD) is estimated to be between 4.90 and 7.08 million [3]. CKD is usually diagnosed in the later stages of disease. The early stage of CKD is independently associated with an increased risk of all-cause mortality and cardiovascular disease (CVD).

According to the China Kidney Disease Network (CK-NET) 2016 Annual Data Report evaluation of hospitalized adult patients, patients with CKD accounted for 4.86% of the total hospitalized population [4]. The age-standardized prevalence of CKD was 7,180/100,000, demonstrating a decrease of 6.1% since 1990 [1]. The onset of CKD might be masked until it is discovered during screenings or in the advanced stages of the disease. The advanced stages of the disease increase the cost of diagnosis and treatment, posing a challenge to the healthcare system.

Some comorbidities, including hypertension, diabetes mellitus (DM), mineral and bone disorder (MBD), renal anemia, and CVD, have been shown to occur in CKD patients. DM and hypertension are the leading causes of CKD [5]. In 2018, the prevalence of hypertension and CKD in individuals aged 60 years and older was 7.7% (95% CI: 7.0–8.5) [6]. Data from several prospective cohort studies indicate that the risk of CVD morbidity and mortality is significantly increased in elderly individuals with decreased kidney function [7–9]. Prior studies have shown that compared to non-CKD status, CKD status is associated with a greater number of comorbidities [10]. Among Medicare beneficiaries, nearly 85% of those with CKD had two or more additional chronic conditions. In contrast, only 37% of individuals without CKD had multimorbidity. Having certain disease conditions may affect the treatment of other comorbidities, and it is possible that comorbid conditions may result in greater costs than those associated with the sum of each single conditions. Considering the impact of comorbidities on spending in CKD patients, it is helpful to synergistically and efficiently address these issues and guide policymaking [11].

In China, the healthcare system consists of government-provided public medical insurance, commercial medical insurance, and self-funded medical services. Public medical insurance consists mainly of three systems: basic medical insurance for urban workers, basic medical insurance for urban residents, and the new rural cooperative medical system. Residents may also purchase commercial medical insurance to obtain more comprehensive medical protection. In addition, some specialized medical services may require out-of-pocket payments. The health insurance payment system is being promoted via measures such as promoting a national pilot program of disease diagnosis-related group (DRG) payments. CKD is an important disease that imposes a substantial burden, and the government has implemented corresponding support measures to alleviate patients’ treatment expenses. During the COVID-19 pandemic, the management of chronic noncommunicable diseases including CKD, was adjusted to adapt to the new context. In our nation, some polices, such as appointment booking, time-slot medical care, “Internet Plus” healthcare and long-term prescriptions, were utilized to manage these patients with chronic noncommunicable diseases.

In this study, we aimed to describe the characteristics, and medication patterns of CKD patients with comorbidities, especially inpatients, as well as the economic costs imposed by their conditions. To address this evidence gap, we conducted comprehensive analyses of real-world data. The primary objectives of this research were to 1) describe the characteristics of inpatients with CKD and comorbidities; 2) describe the healthcare costs for different CKD groups, including the estimated total and average hospitalization costs for inpatients; and 3) analyze the differences in the costs of comorbidities between CKD and other diseases, including MBD, hypertension, DM, renal anemia, and CVD; and 4) describe the patterns of medication usage within each comorbidity group.

## Methods

### Survey Design and Population

The study was a multicenter, nonintervention and retrospective observational study. We gathered a sample of individuals aged 18 and older from the general population. The inclusion and exclusion criteria were set in accordance with the 2012 KDIGO-CKD guidelines [12]. The patients were divided into distinct groups based on whether they underwent dialysis or had other comorbidities, such as MBD, hypertension, renal anemia, DM, or CVD. The data were obtained from hospitals in residential areas.

The patients were diagnosed between March 1, 2020 and August 31, 2021, and on at least one occasion, their information was recorded in the electronic health database. Specifically, individuals who underwent kidney transplant operations were selected for the study. During the postbaseline observation period, patients had at least one all-cause visit. To be included in this study, the patients had to meet at least one of the following diagnostic criteria for CKD [13, 14]:

1) According to the CKD EPI formula, the time interval between any two creatinine tests was between 90 and 730 days, and the eGFR was less than 60 mL/min/1.73 m^2^. The index date was the day when the second abnormal creatinine detection occurred.
2) The time interval between any two routine urine/urinary protein quantitative tests ranged from 90 to 730 days, and the results were all abnormal (i.e., urinary protein; ACR>30 mg/g; AER≥30 mg/24 h; PCR≥150 mg/g; 24 h UP ≥ 150 mg/24 h). The index date was the date when the second abnormal routine urine/urinary protein quantitative test result was detected.
3) The interval between any two routine urine tests was within 90–730 days, and all the results were abnormal (defined as having at least three occasions of high-magnification detection of erythrocytes or BLD≥2+). The index date was the date of the second abnormal urine routine test result.
4) There were 90–730 days between any two diagnosed renal illnesses or those for which an ICD-10 code was assigned. The index date was the day the second diagnosis of kidney disease was made.

The exclusion criteria were as follows: 1) no available age or sex information; 2) simple persistent hematuria without urinary protein, a decreasing eGFR or a CKD diagnosis; and 3) a confirmed diagnosis of infection, calculus, cancer or injury to the ureter, urinary bladder, or urethra before or within 2 months of abnormal hematuria results.

### Data Sources

In this study, the data were obtained from the standard front page of the hospitalization electronic medical records at the National Health Medical Data East Center in Xuzhou city, Jiangsu Province, China. This center utilizes a universal health information platform to collect clinical diagnosis and treatment data for 80 million individuals in Jiangsu Province, following a healthcare big data service model that focuses on sharing information, integrating knowledge, and collaborating across multiple disciplines. The data did not include ID card numbers, residential addresses or contact information. Patients with CKD were identified using either a Chinese diagnostic term or the Chinese version of Classification and Codes of Diseases (GB/T 14,396-2016). The relevant information is summarized in Supplementary Appendix S1. Sociodemographic data (e.g., age, sex), personal health history (e.g., hypertension, DM, anemia), and annual CKD-related resource use information were retrieved. All study researchers and staff members completed a training program to become familiar with the study’s methodologies.

### Cost Estimation

We calculated the annual direct costs of CKD and its comorbidities. The data used in the observation period were quantified for each patient and obtained from medical records. The medical costs were calculated based on different groups, including the dialysis/nondialysis group, and different comorbidities, particularly inpatients. Direct medical costs refer to the expenses incurred for medical resources utilized in prevention and treatment of illnesses. These costs include medications, examinations and hospitalization expenses. The average costs per visit were calculated by dividing the total cost of hospitalizations by the total number of visits of hospitalized patients. The average hospitalization costs were calculated by dividing the total cost of hospitalizations by the total person-years of inpatients. The costs included drug use, bed costs, material costs, inspection costs, laboratory costs, nursing costs, surgical procedures, diagnostic tests, and other expenses. The analysis of comorbidities focused on CKD and other disorders, including MBD, hypertension, DM, renal anemia, and CVDs. During the observation period, we identified the number and proportion of patients who had each comorbidity, as well as their healthcare expenses, among the CKD population.

### Statistical Analysis

We calculated the expenditures of patients with CKD by different comorbidity groups. After applying the selection criteria, 29,660 participants who were 18 years or older were considered eligible for inclusion in the present analyses. Continuous variables are presented as the mean ± standard deviation (SD) or median and interquartile range (IQR), depending on the normality of the data distribution. Categorical variables are presented as frequencies and proportions. Stratification and descriptions of relevant traits are provided. Due to the comorbidity classifications resulting from considering CKD with only one other disease, there may be overlap among patients in each subgroup, rendering the groups nonindependent. Therefore, the appropriate statistical tests were not conducted. All analyses were conducted using SAS software (Version 9.4 SAS Institute, Cary, NC).

### Role of Funding Source

The funders of the study had no role in the design or conduct of the study, including data collection, management, analysis, or interpretation of the results; preparation, review, or approval of the manuscript; or the decision to submit the manuscript for publication.

## Results

### Demographic Characteristics

The information of 29,660 patients was analyzed in the present study. In the group with different comorbidities, the majority of the participants were middle aged (45–65 years), were men (over 50%), and had medical insurance (over 90%). In the present study, only one patient underwent a kidney transplant operation. There were 594 patients on peritoneal dialysis and 2,293 on hemodialysis. Among patients with CKD, glomerulonephritis (GN) was the primary cause of CKD comorbidities in patients with an identified etiology, while in addition to other causes (unclassified kidney disease), GN and DM accounted for 33% of the etiology in the CKD-DM group. A total of 2,631 people suffered major adverse cardiovascular events (MACEs), including 23 deaths. In the different comorbidity groups, more than 10% of people experienced MACEs, and approximately 9.2% of people in the CKD-CVD group experienced MACEs. Heart failure and myocardial infarctions were the most common conditions in the CKD-MBD and CKD-renal anemia groups. Heart failure and nonfatal strokes were the most common conditions in the CKD-hypertension, CKD-DM and CKD-CVD groups (Table 1).

**TABLE 1:** Characteristics of study participants with comorbidities (Economic Burden of Chronic Kidney Disease, Xuzhou, China, 2023).

Characteristics	Total N = 29,660	CKD-MBD N = 820	CKD-hypertension N = 17,829	CKD-renal anemia N = 3,576	CKD-DM N = 7,568	CKD-CVD N = 25,935
Sex (N, %)						
Women	14,517 (48.9)	363 (44.3)	8,451 (47.4)	1,479 (41.4)	3,298 (43.6)	12,869 (49.6)
Men	15,143 (51.1)	457 (55.7)	9,378 (52.6)	2,097 (58.6)	4,270 (56.4)	13,066 (50.4)
Age, y (N, %)						
18 years∼	4,963 (16.7)	257 (31.3)	2,153 (12.1)	815 (22.8)	781 (10.3)	4,128 (15.9)
45 years∼	13,199 (44.5)	374 (45.6)	7,808 (43.8)	1,694 (47.4)	3,527 (46.6)	11,266 (43.4)
65 years∼	6,851 (23.1)	132 (16.1)	4,604 (25.8)	685 (19.2)	1,995 (26.4)	6,195 (23.9)
75 years∼	4,647 (15.7)	57 (7.0)	3,264 (18.3)	382 (10.7)	1,265 (16.7)	4,346 (16.8)
Etiology^a^ (N, %)						
PKD	157 (0.5)	2 (0.2)	108 (0.6)	50 (1.4)	22 (0.3)	128 (0.5)
HTN	210 (0.7)	2 (0.2)	181 (1.0)	10 (0.3)	50 (0.7)	210 (0.8)
ON	810 (2.7)	5 (0.6)	525 (2.9)	29 (0.8)	270 (3.6)	649 (2.5)
GN	3,231 (10.9)	66 (8.0)	2,186 (12.3)	233 (6.5)	1,140 (15.1)	2,721 (10.5)
RCC	426 (1.4)	3 (0.4)	277 (1.6)	11 (0.3)	158 (2.1)	363 (1.4)
DM	1,376 (4.6)	14 (1.7)	555 (3.1)	97 (2.7)	1,376 (18.2)	716 (2.8)
Others	23,450 (79.1)	728 (88.8)	13,997 (78.5)	3,146 (88.0)	4,552 (60.1)	21,148 (81.5)
Medical insurance (N, %)^b^						
Yes	26,946 (92.7)	706 (91.2)	16,411 (93.5)	3,056 (88.6)	6,789 (91.6)	23,918 (93.8)
No	2,126 (7.3)	68 (8.8)	1,132 (6.5)	395 (11.4)	619 (8.4)	1,585 (6.2)
MACEs^c^	2,631 (8.9)	101 (12.3)	1845 (10.3)	581 (16.2)	940 (12.4)	2,394 (9.2)
All-cause death	23 (0.1)	1 (0.1)	11 (0.1)	6 (0.2)	10 (0.1)	19 (0.1)
Hospitalizations due to heart failure	1,185 (4.0)	40 (4.8)	861 (4.8)	247 (6.7)	438 (5.7)	1,075 (4.1)
Nonfatal myocardial infarctions	537 (1.8)	53 (6.4)	332 (1.8)	287 (7.8)	191 (2.5)	407 (1.6)
Nonfatal strokes	1,171 (3.9)	16 (1.9)	854 (4.7)	133 (3.6)	420 (5.5)	1,161 (4.4)

Notes: a: PKD, polycystic kidney disease; HTN, hypertensive nephropathy; ON, obstructive nephropathy; GN, glomerulonephritis; RCC, renal cell carcinoma; DM, diabetes mellitus; Others, CKD, due to other reasons/unclassified kidney disease.

b: missing data.

c: MACEs, major adverse cardiovascular events.

### Annual Costs Associated With CKD

Costs are presented based on disease severity and various comorbidities. The total hospitalization cost for patients in the CKD-CVD group was over 174 million yuan, and the annual total hospitalization cost for patients in the CKD-renal anemia group was greater than that for patients in the other groups. The CKD-CVD and CKD-renal anemia groups had the highest medical costs among the groups. The average hospitalization cost per visit for the CKD-renal anemia and CKD-MBD groups was 8,674.5 (5,154.3–13,949.6) and 8,182.6 (4,798.2–12,844.7) Yuan, respectively, which was greater than that for the other groups (Table 2).

**TABLE 2:** The hospitalization costs of patients by comorbidities, Yuan, M (Q1, Q3) (Economic Burden of Chronic Kidney Disease, Xuzhou, China, 2023).

Costs	CKD-MBD (N = 820)	CKD-hypertension (N = 17,828)	CKD-renal anemia (N = 3,576)	CKD-DM (N = 7,568)	CKD-CVD (N = 25,935)
Hospitalization total costs	9,669,882	138,012,192.9	54,624,354.9	73,077,640	174,053,696
Drug use	2,690,061.1 (27.8)	3,4,941,773.5 (25.3)	15,837,289.3 (29)	20,906,241.1 (28.6)	44,023,199.3 (25.3)
Hospital bed	4,568 (0)	50,654 (0)	48,739 (0.1)	26,216 (0)	75,024 (0)
Material costs	1,633,713.2 (16.9)	22,293,624.5 (16.2)	8,294,411 (15.2)	11,750,139.9 (16.1)	28,078,354.8 (16.1)
Inspection costs	11,202.6 (0.1)	146,794.2 (0.1)	88,183.5 (0.2)	107,229.9 (0.1)	189,174.9 (0.1)
Laboratory costs	28,548.7 (0.3)	329,062.7 (0.2)	226,819.9 (0.4)	268,586 (0.4)	428,687.9 (0.2)
Nursing costs	173,388.6 (1.8)	1,966,243.8 (1.4)	962,279 (1.8)	1,226,122.1 (1.7)	2,470,324.5 (1.4)
Surgical procedures	567,513.9 (5.9)	7,349,958.8 (5.3)	2,610,998.6 (4.8)	3,787,042.3 (5.2)	9,103,158.1 (5.2)
Diagnostic tests	3,651,935.7 (37.8)	40,330,607 (29.2)	20,456,208.8 (37.4)	23,699,907 (32.4)	49,996,342.9 (28.7)
Others	4,329,772.9 (44.8)	53,463,340.1 (38.7)	24,991,339.7 (45.8)	31,389,216.8 (43)	67,054,882.7 (38.5)
Total costs	65,754,445	326,310,436.3	222,647,495.5	150,610,599.7	388,309,181.5
Emergency costs	166,791.1	1,316,190	731,844.6	819,256.7	1,565,437.2
Outpatient expenses	55,917,772	186,982,053.3	167,291,296	76,713,703.3	212,690,048.4
Hospitalization expenses	9,669,882	138,012,192.9	54,624,354.9	73,077,639.7	174,053,696
Total costs annual	59,052	16,235	49,890.8	18,298	13,769.3
Emergency costs	149.8	65.5	164	99.5	55.5
Outpatient expenses	50,218	9,303	37,486.6	9,320.1	7,541.9
Hospitalization expenses	8,684.2	6,866.6	12,240.2	8,878.3	6,171.9
Average cost per visit					
Emergency costs	40 (12–202.3)	75 (17.5–284)	92.4 (18.3–367.5)	92.5 (19–346)	78 (18–290.4)
Outpatient expenses	174.1 (26–844.5)	98.2 (40.8–253)	333.5 (64–2,380.9)	130.4 (46.6–307.8)	92 (41–233.5)
Hospitalization expenses	8,182.6 (4,798.2–12,844.7)	5,292.1 (3,297.8–10,283.9)	8,674.5 (5,154.3–13,949.6)	7,044.7 (3,902.4–12,417.9)	5,087.7 (3,220.5–9,947.4)

**FIGURE 1:**
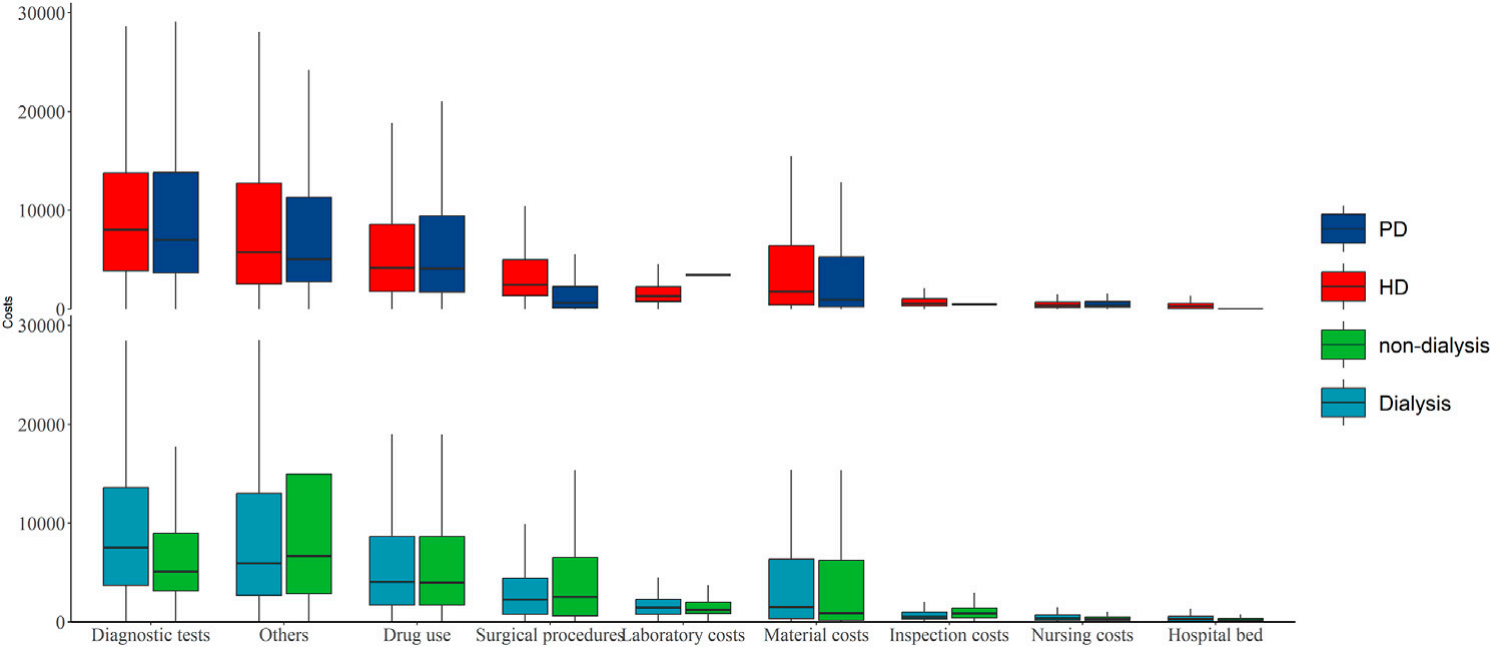
The average hospitalization costs per patient by dialysis and nondialysis [Yuan, M (Q1, Q3); Economic Burden of Chronic Kidney Disease, Xuzhou, China, 2023]. Note: PD: peritoneal dialysis; HD: hemodialysis.

**FIGURE 2:**
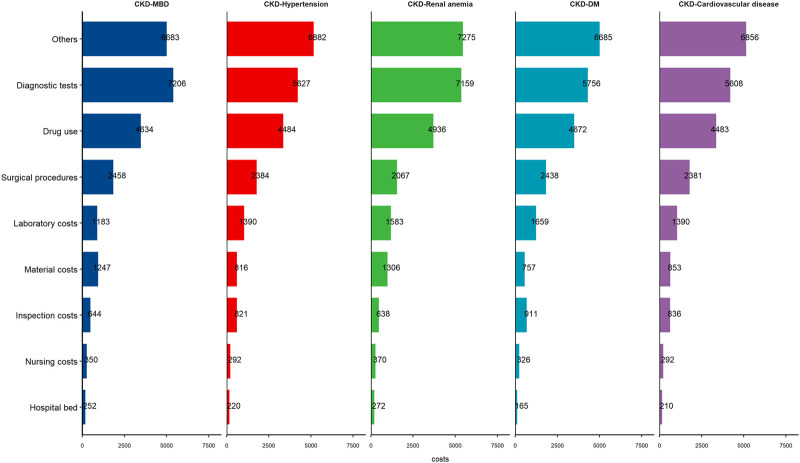
The *per capita* cost of the patients stratified by chronic kidney disease comorbidity groups (Economic Burden of Chronic Kidney Disease, Xuzhou, China, 2023).

The average hospitalization costs were more than two times greater for dialysis patients than for nondialysis patients (17,050.3 (8,150.4–32,730.5) vs. 7,703.9 (3,748.5–18,917.1) Yuan, respectively), and the cost for hemodialysis (HD) patients was greater than that for peritoneal dialysis (PD) patients in the dialysis group (18,107.1 (8,902.4–33,553.7) vs. 13,915.9 (7,125.2–31,658.1) Yuan, respectively). The average hospitalization costs per visit were greater for the dialysis patients than for the nondialysis patients (8,356.1 (5,139–13,737.9) vs. 4,977.9 (3,146.1–10,047.8) Yuan) and the costs for the HD patients were also greater than those for the other groups (8,640.3 (5,325–13,940.8) vs. 7,760.2 (4,972.6–13,341.4) Yuan, respectively) (Figure 1; Supplementary Table S1). Hospitalization costs were substantial for each comorbidity group, with diagnostic tests and drug use costs accounting for more than half of the total costs (Table 2), and the average cost of hospitalization showed similar results (Figure 2; Supplementary Table S2). The average *per capita* costs of the CKD comorbidity patients in the dialysis and nondialysis groups are shown in Supplementary Table S3.

### Patterns of Medications

In total, the proportions of patients taking CKD-related medications, CVD medications, antihyperglycemic agents, lipid-lowering drugs, and antianemia drugs were 60.9%, 53.5%, 23.0%, 25.0%, and 19.2%, respectively. In each comorbidity group, excluding CKD-related drugs, the majority of patients (over 50%) used CVD-related medications. In the CKD-DM group, 64.9% of the patients used antihyperglycemic agents. The most prevalent CKD-related medication categories were angiotensin converting-enzyme inhibitor (ACEIs)/angiotensin receptor blockers (ARBs) and glucocorticoids, which account for 33.4% and 28.8%, respectively, of the total. The majority of CVD drugs were calcium-channel blockers, antiplatelet agents and beta-receptor blockers. Similar results were observed in the different comorbidity groups, and to treat CKD, ACEIs/ARBs were the most commonly used drugs. The majority of CKD-MBD patients used vitamin D and its analogs. Anemia treatment drugs were used by the majority of patients with CKD-renal anemia (Table 3).

**TABLE 3:** Patterns of medications in patients by comorbidities (Economic Burden of Chronic Kidney Disease, Xuzhou, China, 2023).

Drugs	Total	CKD-MBD (N = 820)	CKD-hypertension (N = 17,829)	CKD-renal anemia (N = 3,576)	CKD-DM (N = 7,568)	CKD-CVD (N = 25,935)
CKD-related (N, %)	18,069 (60.9)	594 (72.4)	11,989 (67.2)	2,727 (76.3)	5,096 (67.3)	15,871 (61.2)
ACEIs/ARBs	9,914 (33.4)	421 (51.3)	8,224 (46.1)	1,616 (45.2)	3,121 (41.2)	8,989 (34.7)
Glucocorticoids	8,548 (28.8)	187 (22.8)	4,840 (27.1)	833 (23.3)	1,671 (22.1)	7,835 (30.2)
Immunosuppressants	912 (3.1)	34 (4.1)	613 (3.4)	155 (4.3)	315 (4.2)	742 (2.9)
SGLT-2is	1,485 (5)	24 (2.9)	983 (5.5)	106 (3)	1,252 (16.5)	1,131 (4.4)
Keto acid agents	799 (2.7)	66 (8)	593 (3.3)	353 (9.9)	247 (3.3)	651 (2.5)
Metabolic acidosis	2,348 (7.9)	214 (26.1)	1,467 (8.2)	1,223 (34.2)	691 (9.1)	1767 (6.8)
Kidney-protecting Chinese medications	4,717 (15.9)	212 (25.9)	3,337 (18.7)	983 (27.5)	2,133 (28.2)	3,839 (14.8)
Others	18,720 (63.1)	695 (84.8)	13,211 (74.1)	3,016 (84.3)	5,938 (78.5)	16,185 (62.4)
CVD drugs	15,878 (53.5)	640 (78)	11,901 (66.8)	2,917 (81.6)	4,752 (62.8)	14,072 (54.3)
Diuretics	4,046 (13.6)	215 (26.2)	2,968 (16.6)	1,150 (32.2)	1,480 (19.6)	3,509 (13.5)
Beta blockers	5,846 (19.7)	404 (49.3)	4,485 (25.2)	1,573 (44)	1935 (25.6)	5,105 (19.7)
Calcium-channel blockers	9,690 (32.7)	500 (61)	7,926 (44.5)	2,201 (61.5)	2,989 (39.5)	8,653 (33.4)
Antiplatelet agents	8,454 (28.5)	317 (38.7)	6,392 (35.9)	1,206 (33.7)	3,043 (40.2)	7,709 (29.7)
Anticoagulants	3,278 (11.1)	376 (45.9)	2085 (11.7)	1739 (48.6)	1,027 (13.6)	2,418 (9.3)
Antihyperglycemic Agents	6,836 (23)	204 (24.9)	4,635 (26)	848 (23.7)	4,914 (64.9)	5,413 (20.9)
Metformin	3,641 (12.3)	61 (7.4)	2,629 (14.7)	151 (4.2)	2,512 (33.2)	3,063 (11.8)
Sulfonylureas	1877 (6.3)	36 (4.4)	1,407 (7.9)	80 (2.2)	1,220 (16.1)	1,616 (6.2)
ɑ-glucosidase inhibitors	1752 (5.9)	38 (4.6)	1,260 (7.1)	129 (3.6)	1,372 (18.1)	1,451 (5.6)
Glinides	849 (2.9)	26 (3.2)	595 (3.3)	105 (2.9)	682 (9)	677 (2.6)
Thiazolidinediones	334 (1.1)	3 (0.4)	228 (1.3)	19 (0.5)	249 (3.3)	253 (1)
GLP-1 RA	123 (0.4)	0 (0)	65 (0.4)	6 (0.2)	116 (1.5)	71 (0.3)
Insulin	3,112 (10.5)	147 (17.9)	2005 (11.2)	665 (18.6)	2,565 (33.9)	2,301 (8.9)
Lipid-lowering drugs	7,428 (25)	270 (32.9)	5,576 (31.3)	878 (24.6)	2,834 (37.4)	6,840 (26.4)
Statins	7,157 (24.1)	262 (32)	5,398 (30.3)	864 (24.2)	2,769 (36.6)	6,599 (25.4)
Fibrates	460 (1.6)	36 (4.4)	360 (2)	43 (1.2)	150 (2)	427 (1.6)
Others	183 (0.6)	4 (0.5)	130 (0.7)	21 (0.6)	66 (0.9)	164 (0.6)
Uric acid-lowering agents	1728 (5.8)	97 (11.8)	1,202 (6.7)	644 (18)	576 (7.6)	1,367 (5.3)
Antianemia drugs	5,686 (19.2)	382 (46.6)	3,739 (21)	1,642 (45.9)	1,786 (23.6)	4,772 (18.4)
phosphorus-lowering drugs	1,961 (6.6)	350 (42.7)	1,180 (6.6)	1,290 (36.1)	477 (6.3)	1,319 (5.1)
vitamin D and analogs	3,969 (13.4)	527 (64.3)	2,639 (14.8)	1,705 (47.7)	1,303 (17.2)	3,008 (11.6)
Calcium-sensitive receptor agonists	631 (2.1)	163 (19.9)	349 (2)	418 (11.7)	104 (1.4)	410 (1.6)
potassium-lowering drugs	11 (0)	1 (0.1)	7 (0)	10 (0.3)	7 (0.1)	7 (0)

## Discussion

In the present study, a total of 29,660 patients with CKD were enrolled from Xuzhou city, China. For these patients, we collected and analyzed data from the front page of the electronic medical records. Our research findings indicate that the economic burden of CKD is significant, especially in individuals with CKD and comorbidities. The disease that incurred the highest medical costs within the population was CKD-CVD, while CKD-renal anemia had the highest *per capita* medical cost.

In our analysis, GN and DM accounted for more than 15% of the etiology in patients with CKD and comorbidities, and GN constituted the majority of that proportion. A recent study estimated that the proportion of inpatients with CKD increased from 3.58% in 2010 to 4.95% in 2017. In 2019, the global number of patients with incident CKD due to GN increased by 77% to a total of 606,300 patients (95% UI, 560,100–658,100), accounting for 3% of all CKD patients [15]. Since 2011, the percentage of people with CKD caused by DM has been higher than the percentage caused by GN [16], and the disparity has steadily grown. In 2017, 1.14% of patients admitted to the hospital had CKD-DM, while 0.68% of patients had CKD-GN [17]. According to the China Kidney Disease Network (CK-NET) 2016 Annual Data Report, the most common causes of CKD were DM (26.70%), HTN (21.39%), obstructive nephropathy (ON, 16.00%), and GN (14.41%) [4]. These numbers are not consistent with the studies, which could be partially attributable to regional differences in disease prevalence. The population in our study also had a greater percentage of patients with ON. This finding was consistent with the observation that South China has a greater proportion of patients with ON [18].

We found that the average hospitalization cost of the inpatients in the dialysis group was 17,050.3 (8,150.4–32,730.5) Yuan, including 13,915.9 (7,125.2–31,658.1) Yuan for PD and 18,107.1 (8,902.4–33,553.7) Yuan for HD, which was lower than £20,078 for PD and £24,043 for HD in England [19]; therefore, it is apparent that controlling the progression of disease, even in individuals with early stages of CKD, would be a more effective way to reduce medical costs and alleviate the burden on the healthcare system [20]. According to the data from the United States Centers for Disease Control and Prevention, hospitalization costs account for approximately one-third of the total costs [21]. Nichols and colleagues [22] analyzed the data and reported that inpatient costs accounted for 33.2% of the total costs for all patients. Those results were consistent with the results of our study, suggesting that the cost structure we observed in our data was regionally representative. The published literature has indicated that prior hospitalization burden is an important risk factor for future 30-day readmissions [23, 24].

The actual costs of inpatient care in different studies are difficult to compare due to variations in dialysis cost components and medical insurance coverage. Additionally, the criteria for patient selection differed between studies. In the present study, we aimed to describe the status of hospitalization costs while considering dialysis and comorbidity statuses. The cost components analyzed in this study were more comprehensive and detailed.

Patients with CKD often have DM, CVD, or hypertension. All of these conditions are known to be associated with significant medical costs [25, 26]. Our analyses provide significant data on the comparative costs of CKD patients with these comorbidities, as well as comparisons across different disease stages. The hospitalization total costs of the CKD-hypertension and CKD-CVD groups were greater than those of the other groups; perhaps in these groups, the incidence of these diseases was substantial. In 2018, a survey including a total of 179,873 permanent residents aged 18 years and older was conducted, and a multistage stratified cluster random sampling method was utilized. The results indicated that the prevalence of hypertension was 27.5% (95% CI: 26.6%–28.4%) [27]. CKD-MBD and CKD-renal anemia were associated with particularly higher hospitalization costs *per capita*. The expenses for these patients were double the expenses for patients in the CKD-CVD group. The most significant portion of the expenses for each CKD comorbidity was related to diagnostic test expenses. We cannot determine the temporal order between CKD and the comorbidities we examined from our analysis. It is possible that effective treatment for CKD would impact the costs associated with other diseases. Some costs might be attributed to the progression of new comorbidities or CKD. Some studies have shown that patients with CKD are at increased risk of CVD, which often leads to severe conditions such as heart failure [28].

The treatment of comorbidities needs to involve interdisciplinary approaches. Bao and colleagues [29] collected data from electronic hospitalization summary reports in 38 grade 3A hospitals and found that prescribed drugs were the major contributor to the average hospitalization cost per visit, accounting for 36.2% of the total cost. In that study, the complications with the greatest contributions to the increase in hospitalization costs were CVD and DM, which cost 10,111 and 9,972 Yuan, respectively. That finding is not in accordance with our analysis results. Our data came from a single integrated health system that collected demographic and diagnostic information based on real-world investigation data. Moreover, the patients were not limited to those who visited tertiary hospitals.

The prevalence of CKD among patients with DM was approximately 36%, while it was 31% in individuals with hypertension in the United States [30]. In our nation, the prevalence of hypertension and DM among individuals aged 18 and older were 27.5% and 12.4%, respectively [27, 31]. It is important to control the progression of diseases and comorbidities. To reduce costs, three aspects need attention: 1) the development of CKD in people at risk; 2) the progression from CKD to ESKD; and 3) other chronic conditions, including type 2 DM and heart disease [32].

This study was subject to several limitations. First, due to variations in the level of informatization, certain patients’ electronic medical information was not documented. Since the patients were from one center and convenience sampling was used, the enrolled patient sample may not be perfectly representative of the broader population in Xuzhou city. Second, our study data were classified based only on the presence of comorbidities in the CKD group and not based on the eGFR. Third, some relevant cost items were not distinguished and were thus calculated repeatedly, especially in terms of material costs and inspection costs. Fourth, our investigation lacked information on indirect costs associated with CKD and comorbidities, including expenses related to patients and caregiver productivity loss. The former represents a significant indirect economic burden and quantifying the costs of patient loss of productivity during hospitalization is challenging. Finally, owing to the period of study during the COVID-19 pandemic, the effect of the pandemic may have had an effect on the admission rate and number of inpatients with CKD and other noncommunicable diseases.

Overall, the results of the present study demonstrated that CKD is an important public health issue. The presence of CKD and comorbidities emphasizes the importance of implementing public health strategies aimed at detecting and preventing these conditions in the general population. With the aging population, our nation may experience a greater economic burden related to CKD.
