# Supplementary material for: Analysis of the Economic Burden of Chronic Kidney Disease With Comorbidities Among Patients in Xuzhou, China
Source: Int J Public Health. 2024 Jul 4;69:1607000. doi: 10.3389/ijph.2024.1607000 (PMC11254622; doi:10.3389/ijph.2024.1607000)
Supplement: Supplementary file 1 [file DataSheet1.docx]

**Analysis of the economic burden of chronic kidney disease with comorbidities among patients in Xuzhou, China**

**Table of contents**

Appendix 1. definition of disease

Table S1. The average hospitalization costs per patient by dialysis and non-dialysis group, Yuan, M(Q1, Q3) (Economic Burden of Chronic Kidney Disease, Xuzhou, China, 2023)

Table S2. The hospitalization costs of patient by comorbidities, Yuan, M(Q1, Q3) (Economic Burden of Chronic Kidney Disease, Xuzhou, China, 2023)

Table S3. Average costs per capita of the CKD-comorbidity patients in the dialysis and non-dialysis groups, Yuan, M(Q1, Q3) (Economic Burden of Chronic Kidney Disease, Xuzhou, China, 2023)

Appendix 1. Definition of disease

In the study, comorbid conditions were defined as follows.

Diabetes was defined as use of medications to treat diabetes, history of diabetes (self-report or retinopathy), or, in the detection of the time interval, fasting blood glucose level ≥ 7.0 mmol/L or non-fasting blood glucose level ≥11.1 mmol/L as measured in the laboratory tests.

Hypertension was defined as use of medications to treat hypertension, history of hypertension (self-report) or by blood pressure measurements obtained during screening of systolic blood pressure ≥140 mmHg or diastolic blood pressure ≥ 90 mmHg.

Renal anemia is defined as a combination of anemia in CKD patients with serum creatinine ≥176μmol/L in addition to other causes of anemia, the degree of which is positively correlated with the degree of renal impairment in nephropathic patients.

CKD-MBD is a systemic disorder of mineral and bone metabolism in CKD patients. It includes abnormalities in calcium, phosphorus, parathyroid hormone and/or vitamin D metabolism, bone turnover, mineralization volume, linear growth and strength abnormalities, and vascular or other soft tissue calcification.

Cardiovascular disease conditions include diseases such as heart attack, coronary heart disease, coronary artery bypass graft, angioplasty, angina/angina pectoris, congestive heart failure, atherosclerotic heart disease and stroke.

The detailed classification disease codes can be referred to *China Classification and codes of diseases (GB/T 14396-2016)^1^.*

***Reference:***

*1.* *National Health Commission of the People's Republic of China. Classification and codes of diseases GB/T 14396-2016[EB/OL]. [2023-11-01]. https://openstd.samr.gov.cn/bzgk/gb/newGbInfo?hcno=8127A7785CA677952F9DA062463CBC41.*

Table S1. The average hospitalization costs per patient by dialysis and non-dialysis, Yuan, M(Q1, Q3) (Economic Burden of Chronic Kidney Disease, Xuzhou, China, 2023)

| variable | Dialysis group | | |  | Non- dialysis group(N=55580) |
| --- | --- | --- | --- | --- | --- |
|  | PD (N=594) | HD (N=2293) | Total (N=3071) |  |  |
| **Total costs** | 13915.9(7125.2~31658.1) | 18107.1(8902.4~33553.7) | 17050.3(8150.4~32730.5) |  | 7703.9 (3748.5~18917.1) |
| Drug use | 4099.1 (1722.5~9447.3) | 4198.9 (1781~8599.9) | 4057.5(1722.2~8638.6) |  | 3985.4 (1729.9~8625.2) |
| Hospital bed | 0 (0~0) | 295 (55~573.8) | 280 (55~567.5) |  | 147.5 (45~330) |
| Material costs | 924.8 (236.3~5280.1) | 1774.1 (438.1~6452) | 1494.1 (337.9~6357.8) |  | 860.2 (179.5~6247.8) |
| Inspection costs | 498 (498~498) | 533.5 (312.1~1040.7) | 515.5 (317.5~999) |  | 837.2 (384.2~1421.2) |
| Laboratory costs | 3444.5 (3444.5~3444.5) | 1317.2 (770.6~2278.8) | 1431 (782.4~2279.9) |  | 1218.7 (844.4~1991) |
| Nursing costs | 357 (204.4~754.8) | 369.6 (167.9~700.8) | 359.4 (168~700.8) |  | 268.8 (153.3~496.4) |
| Surgical procedures | 622 (132~2302) | 2458 (1348~4994.5) | 2267 (778~4432.3) |  | 2529.8 (617~6509) |
| Diagnostic tests | 7005 (3682.5~13846.2) | 8024.2 (3872.6~13781.4) | 7519 (3689.1~13611.5) |  | 5083.1 (3123.5~8964.9) |
| Others | 5053.1 (2760~11334.6) | 5776 (2539.4~12748.7) | 5918.3 (2668.7~13018) |  | 6649.6 (2860.6~14970.4) |

Note: PD: peritoneal dialysis; HD: hemodialysis;

Table S2. The hospitalization per capita costs of patient by comorbidities, Yuan, M(Q1, Q3) (Economic Burden of Chronic Kidney Disease, Xuzhou, China, 2023)

| **Drugs** | CKD-MBD  (N=820) | CKD-Hypertension  (N=17828) | CKD-Renal anemia  (N=3576) | CKD-DM  (N=7568) | CKD-Cardiovascular disease (N=25935) |
| --- | --- | --- | --- | --- | --- |
| **Hospitalization costs per capita** | 16374.3(8773.3~32592) | 9011.5 (4418.9~21263.1) | 16954.7(8729.7~33309.1) | 12436.4(6418.4~26110.9) | 8301.9(3975.9~19752.2) |
| Drug use | 4634.3 (2226.4~9281.2) | 4483.9 (2088.3~9054.3) | 4935.9 (2191.5~10129.6) | 4672.3 (2158.4~9509) | 4483.2 (2076.2~9249.9) |
| Hospital bed | 252.5 (59.8~535) | 220 (49~505) | 272.5 (55~553.8) | 165 (51~447.5) | 210 (54.5~476.2) |
| Material costs | 1246.9 (234.8~5482.2) | 816.2 (179.7~5061) | 1306.5 (260.8~5230) | 756.6 (208.6~4440.7) | 853 (193.5~5301.3) |
| Inspection costs | 643.5 (291~1251.2) | 821 (423.5~1319.5) | 638.4 (361~1260) | 911 (432.5~1448) | 835.7 (417.5~1362.5) |
| Laboratory costs | 1183.4 (637.9~1660.2) | 1389.8 (905~2272.2) | 1582.6 (921.3~2369.4) | 1658.8 (963~2414.8) | 1389.8 (891~2272.2) |
| Nursing costs | 350.4 (204.4~648.1) | 292 (175.2~539.9) | 369.8 (195.9~681.6) | 325.6 (180~597.8) | 292 (175.2~554.8) |
| Surgical procedures | 2458 (602.2~5569.5) | 2384.2 (604~5886) | 2067 (735~4252) | 2438 (632~5800) | 2380.6 (604~5919.8) |
| Diagnostic tests | 7206 (3965~13371.9) | 5627.4 (3441~10394.6) | 7159.3 (3962.8~13235.1) | 5755.8 (3444.1~10830.6) | 5607.5 (3405.6~10489) |
| Others | 6683.4 (3236.2~14523) | 6881.6 (3083.7~14978.6) | 7275.4 (3223.6~16015.6) | 6684.6 (2940~15055.3) | 6856.2 (3060~15194.3) |

Table S3. Average costs per capita of the CKD-comorbidity patients in the dialysis and non-dialysis groups, Yuan, M(Q1, Q3) (Economic Burden of Chronic Kidney Disease, Xuzhou, China, 2023)

| Variables | CKD-MBD | | CKD-  Hypertension | | CKD-  Renal anemia | | CKD-  DM | | CKD-  Cardiovascular disease | |
| --- | --- | --- | --- | --- | --- | --- | --- | --- | --- | --- |
|  | dialysis | Non- dialysis | dialysis | Non- dialysis | dialysis | Non-dialysis | dialysis | Non-dialysis | dialysis | Non-dialysis |
| Average costs per capita | 17395.3 (9380.4~34318.2) | 13690.8 (7640.8~27458.7) | 17057.9  (8500.5~  33577) | 8479.9  (4143.6~  19243.4) | 18589  (8657.3~  34819) | 15667  (8865~31915.4) | 18747.7 (10085.5~40288.8) | 11846.2  (6089.4~  24692.2) | 17865.4 (8664.5~34685.6) | 7804.3 (3814.6~18165.1) |
| Drug use | 4533.9 (2221.4~9009.7) | 4994.3  (2251~10078.2) | 4335.9  (1832.7~  9088.4) | 4517.4  (2133.3~  9046.5) | 4383  (1842.1~  9067.1) | 5411.9  (2466.3~10988.2) | 5061.5  (2361.2~  11054.6) | 4625.3  (2148.2~  9342.6) | 4380 (1925.8~  9451.6) | 4501.2 (2108~  9234.9) |
| Bed costs | 327.5 (128.8~561.2) | 41.5  (35.8~47.2) | 357.5  (68.2~  646.2) | 160  (42.5~  405) | 320  (55~  580) | 210  (53~  470) | 235  (54~  552.5) | 165  (53~420) | 357.5 (61~646.2) | 170 (45~373.8) |
| Material | 1201.1 (267.9~4914.7) | 1507.4 (227.3~7058.2) | 1319.1  (265.6~  6456.9) | 740.5  (171.3~  4718.9) | 1679.2  (337.9~  6442.8) | 1068.4 (221.1~4492.5) | 1489.3 (428.2~  6473.6) | 666.9 (196~3919.1) | 1337.3 (285.9~6472.9) | 783.5 (185.9~4939.6) |
| Inspection | 750.5 (397.2~1513.8) | 342 (226.5~457.5) | 566  (325~  1120) | 947.6  (567.8~  1409.8) | 541  (319~  1099.4) | 700  (573~1379.4) | 593.5 (413.8~  1147) | 941  (514~1470) | 553.5 (324.2~1089.1) | 907.7 (526~1428) |
| Laboratory | 1547.8 (834.5~1895.6) | 626.6 (123.8~1098.8) | 1388.7  (737.4~  2123) | 1389.8  (961.8~  2288.7) | 1258.6  (794.2~  2278.8) | 1977.3 (1005.4~2700.7) | 1850 (1609.5~  3291.1) | 1464 (919.5~2348.9) | 1388.7 (737.4~2173.2) | 1389.8 (957.9~2288.7) |
| Nursing | 350.4 (204.4~659.2) | 304.6 (197.3~635.9) | 350.4  (160.4~  648.1) | 288  (175.2~  521.3) | 369.6  (164.3~  700.8) | 374.4  (204.4~648.5) | 406  (194.1~  798.7) | 321.2 (178.6~572.7) | 363.7 (175.2~675.3) | 290.8 (175.2~530.6) |
| Surgical | 2458 (826~5481.5) | 2415.7 (380.3~5569.5) | 2167.5  (671.5~  4242.2) | 2416.9 (600~  6158.2) | 2447.2  (975~  4702) | 2016  (553~3894) | 2268 (1122.5~  4420.5) | 2447.2 (601~6006) | 2166 (655~4481.5) | 2401 (600~6158) |
| Diagnostic | 7264.5 (3979.4~13995.2) | 6856.5 (3965~11862.7) | 7882.7 (3689.1~  14145.6) | 5447.1  (3400.7~  9709.9) | 7636.8  (3928.7~  14136.7) | 6882 (4065.6~12518.2) | 8903.9 (4178.8~  16459.2) | 5536.7 (3355.5~9890.1) | 8086.8 (3853.5~14228) | 5428.5 (3369.4~9811.2) |
| Others | 6042.9 (3019.9~13159) | 7506 (3478~16791) | 6340.8 (2726.1~  13967.6) | 6978.5 (3171~  15055.3) | 6361.3  (2786.3~  15340.6) | 8143.9 (3911~16827.2) | 7462.1 (3314.9~  16100.2) | 6476 (2890.9~14834.3) | 6538.6 (2831.7~15239.4) | 6902.4 (3100.4~15193.5) |
